# Ultrafast Anisotropy Decay Reveals Structure and Energy Transfer in Supramolecular Aggregates

**DOI:** 10.1021/acs.jpcb.3c04719

**Published:** 2023-08-18

**Authors:** Vesna Erić, Jorge Luis Castro, Xinmeng Li, Lolita Dsouza, Sean K. Frehan, Annemarie Huijser, Alfred R. Holzwarth, Francesco Buda, G. J. Agur Sevink, Huub J. M. de Groot, Thomas L. C. Jansen

**Affiliations:** † Zernike Institute for Advanced Materials, University of Groningen, 9747 AG Groningen, The Netherlands; ‡ Department of Chemistry and Hylleraas Centre for Quantum Molecular Sciences, University of Oslo, Sem Sælands vei 26, 0315 Oslo, Norway; ¶ Leiden Institute of Chemistry, Leiden University, Einsteinweg 55, 2300 RA Leiden, The Netherlands; § MESA+ Institute for Nanotechnology, University of Twente, Drienerlolaan 5, 7522 NB Enschede, The Netherlands; ∥ Department of Biophysical Chemistry, Max Planck Institute for Chemical Energy Conversion, Stiftstraße 34-36, 45470 Mülheim, Germany

## Abstract

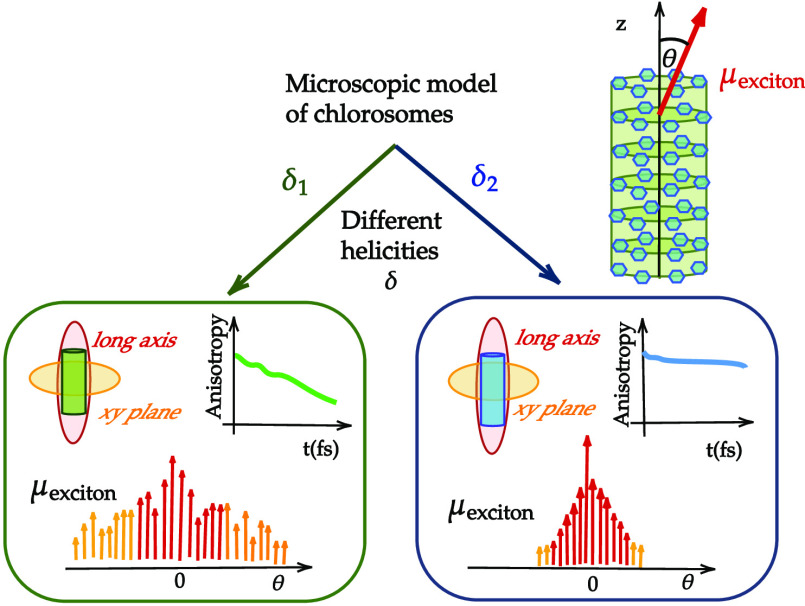

Chlorosomes from green bacteria perform the most efficient light capture and energy transfer, as observed among natural light-harvesting antennae. Hence, their unique functional properties inspire developments in artificial light-harvesting and molecular optoelectronics. We examine two distinct organizations of the molecular building blocks as proposed in the literature, demonstrating how these organizations alter light capture and energy transfer, which can serve as a mechanism that the bacteria utilize to adapt to changes in light conditions. Spectral simulations of polarization-resolved two-dimensional electronic spectra unravel how changes in the helicity of chlorosomal aggregates alter energy transfer. We show that ultrafast anisotropy decay presents a spectral signature that reveals contrasting energy pathways in different chlorosomes.

## Introduction

Green bacteria are anaerobic organisms capable of photosynthetic growth under very low light intensities.^1,2^ They can, for example, survive deep down in the sea^3^ or by utilizing only geothermal energy.^4^ This unique behavior is achieved through optimization of their photosynthetic apparatus, which ensures robust and efficient light harvesting and energy transfer within and between the different structural units.^5−7^ Chlorosomes are the essential organelles responsible for the initial light capture and energy transfer in green bacteria.^8,9^ They consist mainly of self-assembled bacteriochlorophyll (BChl) molecules. The absence of a protein scaffold gives chlorosomes one more unique feature among natural light-harvesting systems. These observations demonstrate the importance of revealing the intricate structure–property relationship in chlorosomes. Since photosynthetic complexes serve as an inspiration for artificial light-harvesting designs for efficient solar energy conversion,^10−15^ we expect that detailed studies of the molecular mechanisms responsible for ultrafast energy transfer in chlorosomes will accelerate further applications.

Inherent heterogeneity in the composition of chlorosomes results in a hierarchy of structural disorder,^16^ and determination of their structure requires the combination of different experimental and theoretical methods. Findings from cryo-EM^17^ indicated that bacteriochlorophyll molecules self-assemble into secondary structures like concentric cylinders and curved lamellae, defining a mesoscopic (long-range) type of disorder.^16^ The spacing between these structures is around 2.1 nm.^17^ Solid-state NMR^18^ provided constraints on the short-range order and showed that BChl *c* molecules form parallel *syn-anti* stacks, which promote self-assembly into cylindrical supramolecular structures.^18^ The stability of the aggregates is ensured through close packing of the chromophores achieved through π–π stacking interaction, coordination of the central magnesium atom with a hydroxy motif of a neighboring chromophore, and hydrogen bonding.^18,19^ Interactions between the neighboring BChl *c* stacks lead to the formation of helical motifs within the aggregates imposing a macroscopic helicity of the whole cylindrical aggregate.^18,20,21^

The close packing of BChl *c* molecules is responsible for the emergence of collective delocalized excitations, denoted as excitons. The excited-state properties depend on the mutual arrangements of the molecules and their transition dipole moments within the aggregates, dictated by the cylindrical geometry and helicity of the aggregates^22^ and the presence of intra-aggregate, molecular scale disorder.^9,16,23,24^ The excitonic states determine the optical response and energy transfer in chlorosomes,^9,25−27^ making them responsible for the optimized function of the aggregate. Exciton states in homogeneous (no disorder) cylindrical J-aggregates have a Bloch form characterized by their longitudinal, *k*_1_, and transverse momentum, *k*_2_.^22^ In such an ideal case, the three dominant optically active states have different directions in their transition dipole moments. All of these states have *k*_1_ = 0. For excitons with *k*_2_ = 0, the transition dipole moment is parallel to the cylinder axis in contrast to the two degenerate states with *k*_2_ = ±1, for which the transition dipole moments are perpendicular to each other and to the cylinder axis.^22^ These states are said to have different polarization parallel, *k*_2_ = 0, and perpendicular, *k*_2_ = ±1, to the cylinder axis. Realistic cylindrical systems with disorder will not host such ideal states. However, similar selection rules will exist, leading to the emergence of multiple *k*_2_ = 0 like and *k*_2_ = ±1 like states along a disordered cylinder.

As previously discussed, the presence of disorder in chlorosomes significantly alters the excitonic landscape and optical properties in chlorosomes. Optical measurements performed on chlorosomes from different bacterial species and comparisons between wild-type and mutants^27^ show significant differences in linear absorption^28,29^ and linear and circular dichroism spectra.^25^ This distinction reveals the variability in structures of chlorosomes from different species.^18,25^ Chlorosomes from mutants contain molecular building blocks with smaller side groups and have prominently narrower spectra^27^ revealing the impact of molecular packing on the spectral broadening.^27^ Additionally, time-resolved measurements allow us to disentangle the time scales that arise in these systems and connect them to the occurrence of processes that reflect the contribution of different scales of disorder in chlorosomes.^30^ All of this supports our aim to create a more realistic description of chlorosomes that includes the effects of molecular scale disorder.^31^

Previously, we established the connection between structural disorder on the scale of individual BChl *c* molecules and found that differences in hydrogen bonding result in a significant broadening of the spectra of individual chlorosomes.^31^ Here, we aim to extend our investigations and show how the helical arrangements of BChl molecules within the chlorosome aggregates alter the spectral response and affect the exciton dynamics in the system, as probed in steady and time-resolved spectroscopy experiments.^9,28,29,32−35^ Our approach will be based on the simulation of linear and two-dimensional electronic spectra (2D ES) of two chlorosome model systems characterized with different macroscopic helicity. The differences between these structures are motivated by recent discussions in the literature proposing these two distinct helicities.^19,20,24,27,36,37^

2D ES is a versatile experimental technique that captures exciton dynamics with femtosecond temporal resolution.^38−40^ It is a third-order optical technique where the signal is acquired after exposing the sample to the interaction with three ultrafast laser pulses.^41^ Two initial pump pulses are separated by a time interval *t*_1_ giving the first coherence time. The system evolves during the waiting time, *t*_2_, before the interaction with the final probe pulse, followed by the detection of the emitted signal after the time interval *t*_3_, that is, the second coherence time. Fourier transforms over the coherence times *t*_1_ and *t*_3_ give the pump (ω_1_) and probe frequencies (ω_3_), respectively. The obtained three-dimensional signal contains information on the dynamics of excited states and is represented as a two-dimensional correlation map between the pump frequency, ω_1_, and the probe frequency, ω_3_, recorded for a fixed waiting time *t*_2_.^41,42^ Photoexcitation with ultrafast broadband pulses leads to coherent excitation of multiple excitons, which is followed by energy transfer via many complex pathways. Hence, 2D ES has been successfully used for characterizing the dynamics of excited states in the light-harvesting antennae,^5,43−46^ including chlorosomes,^9,34,35,47,48^ and for disentangling energy transfer pathways within these complex systems. Control of the observed signals can be achieved using various pulse polarization schemes, like parallel, perpendicular, and cross-polarized.^46,49^ Combining 2D polarization spectra obtained using different polarization schemes with theoretical modeling provides information on the structure of aggregates.^41,50,51^ Thus far, all 2D ES experiments on chlorosomes were reported using a standard parallel polarization scheme.^9,34,35^

Analogous to pump–probe experiments, it is possible to measure anisotropy decay using polarization-resolved 2D ES setups.^41,52^ Such an experiment requires the simultaneous measurements of the 2D ES signal using parallel and perpendicular pulse schemes.^53,54^ 2D ES provides a frequency-resolved 2D map, quantifying properties of individual states, in contrast to pump–probe measurements, which yield the average behavior of all optically active states within the excited exciton band. Dynamics in the system, like rotational motion and energy transfer, will lead to anisotropy decay during the waiting time.^29,55^ Anisotropy decay experiments on chlorosomes from various bacterial species grown under different conditions are performed using pump–probe setups.^28,29,33^ These measurements displayed a puzzling variation in the initial and residual values of the anisotropies as well as in the time scales of the anisotropy decays. Here, we will use simulations of polarization-resolved 2D ES to elucidate the underlying molecular mechanism responsible for the variations in experimentally observed anisotropy decay.^28,29,33^ Additionally, we predict ultrafast anisotropy decay as a spectroscopic signature that distinguishes between chlorosome structures and singles out ultrafast transfer between different types of exciton states present within a single chlorosomal cylinder.^9^

## Methods

This study examines how different arrangements of BChl *c* molecules within chlorosomal aggregates alter the optical response of emergent exciton states. Our approach consists of a first-principles modeling workflow described in refs (31,56). We will adopt the microscopic description of chlorosomes as cylindrical structures^16,19,27,57^ obtained by rolling up a two-dimensional sheet built of parallel *syn-anti* stacks^18^ of BChl *c* molecules, as schematically depicted in the Supporting Information (Figure S1). Here, our starting point is two different supramolecular aggregates, both consisting of three concentric cylinders,^26^ containing 27675 and 27829 BChl *c* molecules, respectively. These two systems differ only in their helicity^21^ quantified by the values of their chiral angle δ = 49.6° and δ = 112.3° that is defining the rolling of the cylinders. The initial structures have been generated using a protocol implemented in software CTubeGen.^21^ Information about the unit cell, and geometric parameters of both model systems are summarized in the Supporting Information (Figure S1 and Table S1). The two model systems will be denoted as Systems 1 and 2 in the remainder of this paper.

We simulate exciton dynamics using a quantum-classical approach within which we describe the motion of nuclei classically and the dynamics of the excited states quantum mechanically.^56^ This framework is already successfully applied in studies of time-resolved two-dimensional spectra, energy transfer, and anisotropy decay in other light-harvesting complexes such as light-harvesting complex 2 (LH2),^58^ the Fenna–Matthews–Olson complex (FMO),^46^ and chlorosomes.^59^ The predicted ultrafast anisotropy decay for LH2^58^ was in very good agreement with the experimental observations.^60^ The advantage of the quantum-classical method is that we can investigate the dynamics of model systems with a realistic size and geometry of extensive molecular aggregates. Additionally, this method is not constrained to harmonic bath dynamics^61^ and allows us to include the effect of anharmonicities often seen in, for example, hydrogen-bonding systems, which can have significant consequences on the two-dimensional spectra.^62,63^ The presence of very delocalized exciton states in chlorosomes^31^ ensures their robustness to perturbations from localized intramolecular vibrations, which require a quantum description^64^ and justifies the approximation of the bath with classical trajectories. Previous studies comparing the applied quantum-classical approach with a full quantum treatment^61^ for model systems, where the full quantum treatment is possible, demonstrate the need to be cautious with the interpretation of thermalization dynamics.^65,66^

Hence, we model the nuclear fluctuations, i.e., bath dynamics, using classical molecular dynamics trajectories obtained by all-atom molecular dynamics simulations in the canonical ensemble at 300 K using GROMACS software.^67^ The interactions between atoms are described using the OPLS-AA^68^ force field. After equilibration for 1 ns, we performed production runs of 10 ps for each starting structure. The structural dynamics of these systems, based on molecular dynamics simulations, is described previously in refs (19−21,26).

**Figure 1:**
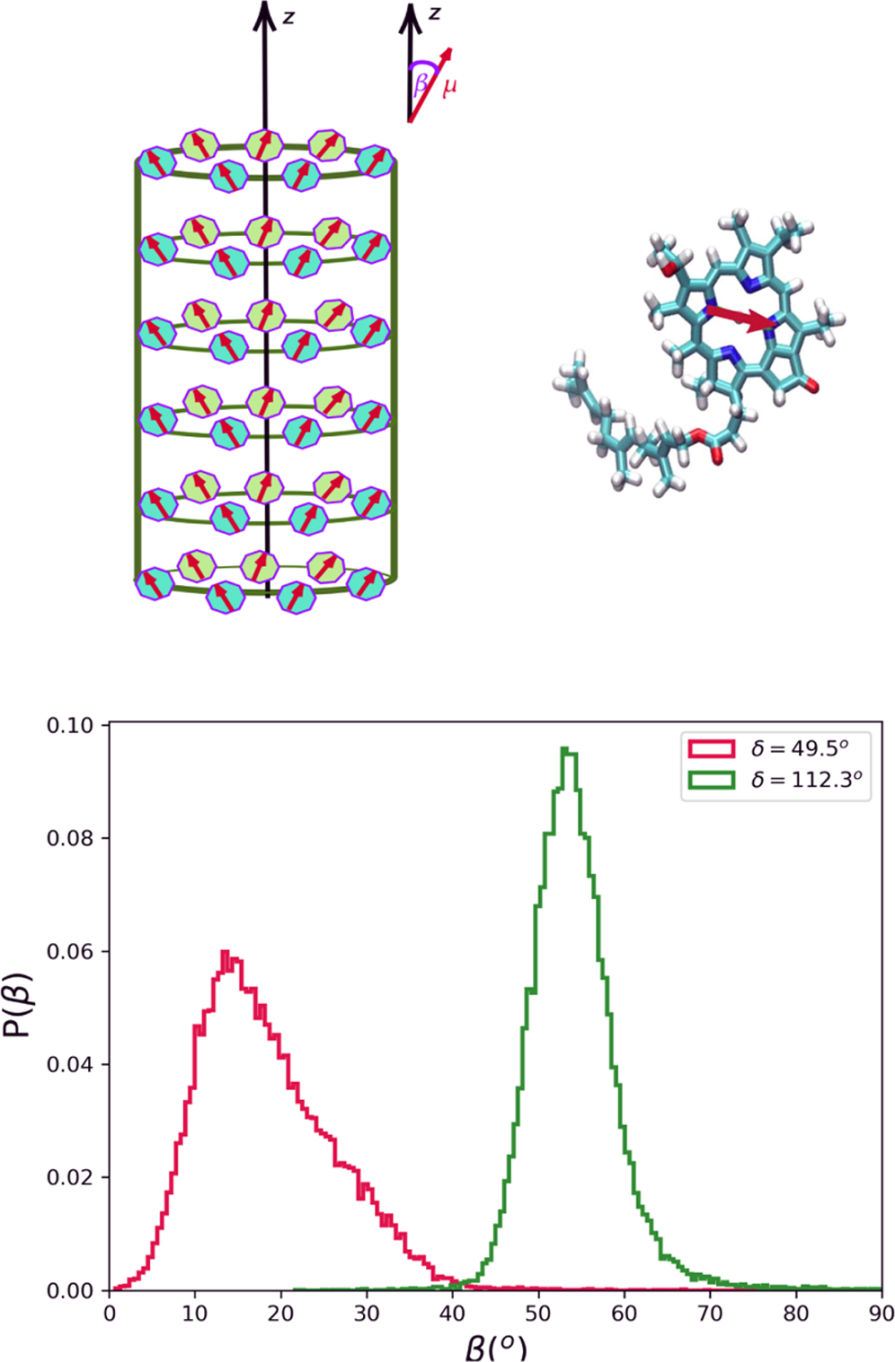
Top: Schematic representation of a single-walled supramolecular cylindrical aggregate and the structure of the BChl *c* molecular building block. The molecular transition dipole moment vectors are shown as red arrows. For every BChl *c*, the transition dipole moment is mapped on its atomic structure as a vector connecting two nitrogen atoms in the porphyrin ring assigned as N_*A*_ and N_*C*_. Bottom: the distributions of β angles, enclosed between the molecular transition dipole moment μ⃗ and the long axis of the aggregate *z⃗*, are shown for System 1 (δ = 49.5°) and System 2 (δ = 112.3°).

We represent the collective electronic excitations, delocalized exciton states dependent on the molecular environment and bath dynamics, with a time-dependent Frenkel exciton Hamiltonian^69^
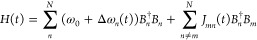
1The summation terms are the transition energies of individual monomers within the aggregate and the excitonic coupling between different monomers, respectively. Our focus is on the *Q*_*y*_ band showing the most prominent peak in the absorption spectra of chlorosomes.^25^ Here, the *Q*_*y*_ exciton states are described within the second quantization formalism,^70^ where *B*_*n*_^†^ and *B*_*n*_ represent the Paulionic creation and annihilation operators for the *Q*_*y*_ excitation of molecule *n*. The Hamiltonian, thus, treats each molecule, and for the manifold of double-excited states, each molecule can only be excited once. Since the BChl *c* molecules are embedded within the aggregate, their transition energy is shifted from the energy of ω_0_ = 15390 cm^–1^ of an isolated molecule.^25^ This energy shift is indicated by the time-dependent term Δ*ω*_*n*_(*t*) determined with a charge density approach.^71,72^ Hence, we account for diagonal disorder by including how the electrostatic environment, represented by partial charges of atoms in neighboring molecules, alters the energy of the excited state, relative to the ground state of the molecule. The ground and excited state partial charges were determined using the CHELPG scheme,^73^ as described in ref (31). The excitonic couplings *J*_*mn*_(*t*), i.e., the interactions that are responsible for the formation of the delocalized excitonic states, are calculated using the point dipole approximation.^20,31^ However, it represents a good choice due to the restrictions coming from the computational cost connected with the large system size. Additionally, previous studies of helical cylindrical aggregates proved the utility and accuracy of this coupling scheme.^20,22,24,31^ The molecular transition dipole moment vectors μ⃗, responsible for excitonic coupling, are mapped onto the conformation of the BChl *c* molecules along the connection of the nitrogen atoms conventionally denoted *N*_*A*_ and *N*_*C*_, as shown in Figure 1. The magnitude of the transition dipole moment is chosen to be 5.48 D.^25^ The complete details on the parametrization of the model Hamiltonian are given in ref (31).

We obtained the field-free evolution of the exciton states by solving the time-dependent Schrödinger equation. Partitioning the dynamics into short time intervals (4 fs), during which we assume a time-independent Hamiltonian, allows us to solve the equation numerically using the numerical integration of the Schrödinger equation (NISE) method.^58,74^ This framework allows for nonadiabatic simulations of large-scale systems under the assumption of the high-temperature limit in which all states in the equilibrium are populated with equal probability, hence, neglecting the energy feedback of the system to the bath. This allows us to use precalculated nuclear trajectories since they are independent of electronic dynamics. The high-temperature limit is a suitable approximation to describe the ultrafast processes that are of our interest.^66^ We calculate the linear and nonlinear optical responses in the perturbative regime using the response function formalism^42^ as implemented in NISE 2017 program.^58,74^ The high computational cost of the 2D ES calculations, due to their scaling^75^as N^3^ with the number of molecules, restricts these simulations to smaller subsystems (see also ref (31).), containing 2639 and 2675 molecules for System 1 and System 2, respectively. Such structures are extracted from the middle tube of the concentric three-tube systems, keeping the parameters as determined for the whole system, following the procedure in ref (31).

We used coherence times in the intervals of [0,128] and [0,196] fs for linear and 2D ES spectra, respectively, which we sampled with the already mentioned timesteps of Δ*t*_1_ = 4 fs. The waiting times were varied up to *t*_2_ = 350 fs with time increments of Δ*t*_2_ = 24 fs. We obtain the linear spectra as a Fourier transform over the two-point transition dipole moment response function^74^ and the 2D ES spectra as 2D Fourier transform over the *t*_1_ and *t*_3_ coherence times.^58^ The total 2D ES spectra are the sum of the rephasing (*k⃗*_*s*_ = −*k⃗*_1_ + *k⃗*_2_ + *k⃗*_3_) and nonrephasing (*k⃗*_*s*_ = *k⃗*_1_ – *k⃗*_2_ + *k⃗*_3_) signals, where *k⃗*_*i*_, with *i* = 1,2,3 are the wave vectors of the three laser pulses and *k⃗*_*s*_ is the wave vector of the emitted signal. Polarization-resolved electronic spectra^53^ represent a combination of the 2D ES spectra obtained from different pulse sequences parallel and perpendicular.^49^ The anisotropy is defined as the difference of the signal intensity *I*_⊥_ obtained using the perpendicular, compared to the signal intensity *I*_∥_ from the parallel pulse scheme, as given in eq 2
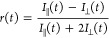
2Our simulations are in the impulsive limit, so we neglect pulse shape effects.^66^ We included effects of homogeneous and inhomogeneous broadening, caused by the presence of mesoscale disorder^16^ on the linear absorption and 2D ES spectra by weighting simulated response functions with exponential (τ_homo_ = 300 fs) and Gaussian (τ_inhomo_ = 166 fs) apodization functions,^49^ as reported previously in ref (31). The time scale responsible for inhomogeneous broadening is based on estimates from hole-burning studies.^23^

## Results and Discussion

We will proceed with the results of our case study of the two microscopic chlorosome model systems (System 1 and System 2) distinct by their chiralities (δ angle). The two models exhibit different distributions of the angle between the molecular transition dipole moment vector μ⃗ and the main axis of the cylinder (*z⃗*), denoted by β, as depicted in Figure 1. As previously shown, this angle has a profound impact on the spectroscopy of cylindrical systems.^22,27,76,77^ These models are in line with the structures of chlorosomes proposed based on cryo-EM studies^20^ suggesting δ ≈ 49.5° (System 1 in our model) and single-molecule spectroscopy^27,36^ reporting values of β ≈ 54° (System 2). The distributions of the β angles, obtained from the MD trajectories of two systems, are shown in Figure 1, and their mean ⟨β⟩ and standard deviation σ_β_ are summarized in Table 1.

**Table 1:** Distributions of β Angles Predicted by the MD Simulations for the Two Microscopic Chlorosome Model Systems (System 1 and System 2)

distribution of β angles		
	⟨β⟩(deg)	σ_β_(deg)
System 1	18	7
System 2	54	5

Both systems show distinct non-Gaussian distributions in the β angles, highlighting the variability in the arrangements of BChl *c* molecules within each aggregate. In System 1, the β distribution is centered around ⟨β⟩ ≈ 18° and very skewed toward higher β angles.

This value is in good agreement with the estimates of 20° based on fluorescence anisotropy measurements on chlorosomes from *Chloroflexus aurantiacus*.^37^ This angle has also been proposed for *Chlorobaculum tepidium*.^20^

For System 2, the distribution of β angles is much narrower and centered around ⟨β⟩ ≈ 54°. Our estimate of standard deviations is in good agreement with the value of 4.8 reported for zinc chlorin aggregates, an artificial nanotube system mimicking chlorosomes, based on the fitting of single-molecule data of *C. tepidium*.^24,36^

**Figure 2:**
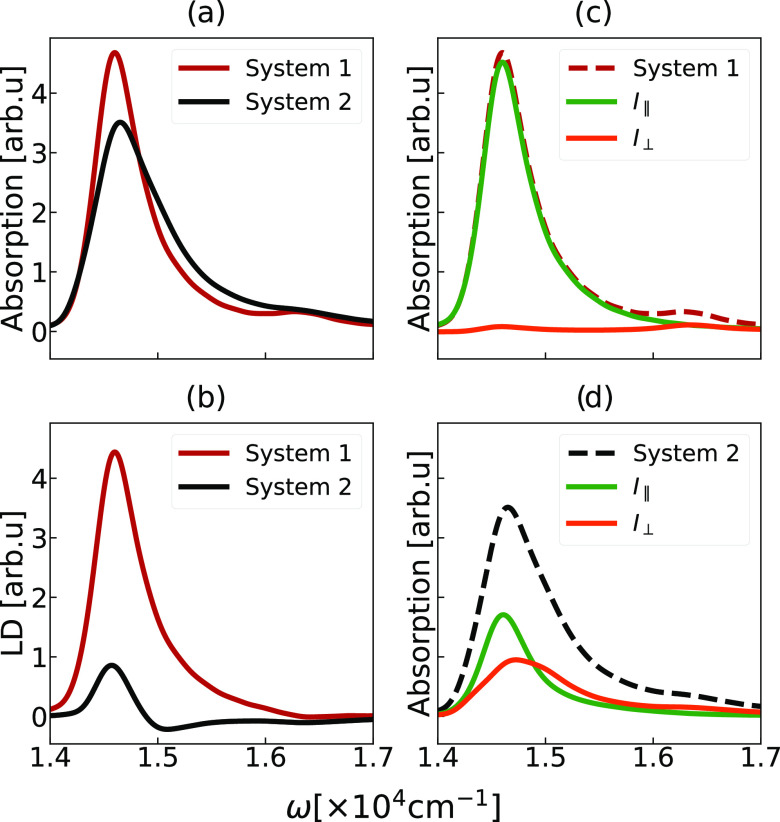
Simulated linear spectra of the two model systems. (a) Linear absorption, (b) linear dichroism spectrum, and (c, d) contributions of parallel (green line) and perpendicular optical components (orange line) to the overall linear response of System 1 (red dashed line) and System 2 (black dashed line), respectively.

We can now assess the relationship between the different arrangements of the molecules within these aggregates with the change in the linear optical response. In Figure 2, we compare the simulated linear absorption and linear dichroism (LD) spectra of the two model systems. Their peak positions and widths are listed in Table 2. Both models capture the non-Gaussian shape of the absorption spectra, characterized by the long tail extending to the high-energy part of the band, as observed experimentally^9,29^ for different chlorosomes. As typically observed for J-aggregates,^70^ the spectra are red-shifted with respect to the molecular transition frequency, due to strong excitonic coupling. Notably, the absorption spectrum of System 2 exhibits the additional broadening compared to System 1, quantified by the 200 cm^–1^ or 40% larger full width half-maximum (fwhm) (see Table 2). Furthermore, System 2 has a more red-shifted spectrum. The absorption spectra of chlorosomes from the green sulfur bacteria *C. tepidium* are more red-shifted and significantly broader than spectra collected from the green nonsulfur bacteria *C. aurantiacus*. The observed differences are, thus, within the natural variation between bacterial species.^25,29,78^ The obtained values of the calculated spectral width are well within the range from 536 to 772 cm^–1^ reported from fluorescence–excitation spectra of a collection of individual chlorosomes from *C. tepidium*.^79^ We provide a direct comparison of our simulated linear absorption spectra of System 1 and System 2 with the experimental measurements on chlorosomes from *C. aurantiacus* and *C. tepidium* in the Supporting Information (Figure S3).

**Table 2:** Parameters Describing the Simulated Linear Absorption Spectra and Components of Transition Dipole Moments of Exciton States Present in Two Model Systems^a^

Linear absorption spectra		
	⟨ω⟩ (cm^–1^)	fwhm (cm^–1^)
System 1	14600	517
System 2	14650	750

Transition dipole moment components		
	*I*_∥_	*I*_⊥_
	⟨ω⟩ [fwhm](cm^–1^)	⟨ω⟩ [fwhm] (cm^–1^)
System 1	14600 [517]	
System 2	14640 [520]	14730 [900]

a In both cases, the same extent of mesoscale disorder^16,23^ is included.

The calculated LD spectra of the two systems, shown in Figure 2b, are significantly different, exposing the contrast in how the two systems absorb light polarized parallel or perpendicular to the long axis of the system. The LD spectrum calculated for System 1 consists of a single positive feature that almost completely corresponds to the shape of the absorption spectra. The LD spectrum of System 2 involves a lower-frequency positive and a higher-frequency negative signal.

To gain better insight, we performed a decomposition of the linear response of the two model systems quantifying the absorption in the direction parallel (*I*_∥_ ≈ ∑_*i*_*M*_*i,z*_^2^) and perpendicular () to the long axis of the system. Here, *M*_*i*,α_ corresponds to the Cartesian component of the transition dipole moment of the excitonic state i. The resulting optical components are presented in Figure 2c,d. The observed features extend the predictions of the idealized models of helical cylindrical aggregates^22,76,77^ and demonstrate how in the presence of realistic molecular disorder, every optical component is comprised of many partially localized exciton states. For System 1, a single optical band *I*_∥_ dominates the absorption spectrum due to the response of the exciton states polarized parallel to the long axis of the cylinder. A small contribution of the perpendicular component *I*_⊥_ is present in the high-energy tail of the spectrum. This finding agrees with the observations of very homogeneous polarization properties within the optically active part of the exciton band of some chlorosomes.^57^

In contrast, the absorption of System 2 defines the presence of two distinct optical components (*I*_∥_ and *I*_⊥_) manifesting significant spectral overlap. This leads to cancellation between the two bands in the overall LD spectrum. With this, we show that the organization of BChl *c* molecules within System 2 leads to a more dispersed distribution of directions of the transition dipole moments of the excitonic states with a mean that significantly deviates from the long axis of the aggregate. Hence this aggregate is optimized for efficient absorption of light traveling from different directions. This feature is likely necessary for organisms living in low-light conditions, like deep in the ocean, interacting predominantly with diffuse light.^3,4^ Therefore, changes in the molecular arrangements within the aggregate can be an important evolutionary mechanism for adaptation to versatile light conditions. Observation of the optical components with different polarization properties^16,27^ of chlorosomes from the green sulfur bacteria *C. tepidium*, known for their efficient photosynthesis in diffuse low-light conditions,^8^ supports our argument.

**Figure 3:**
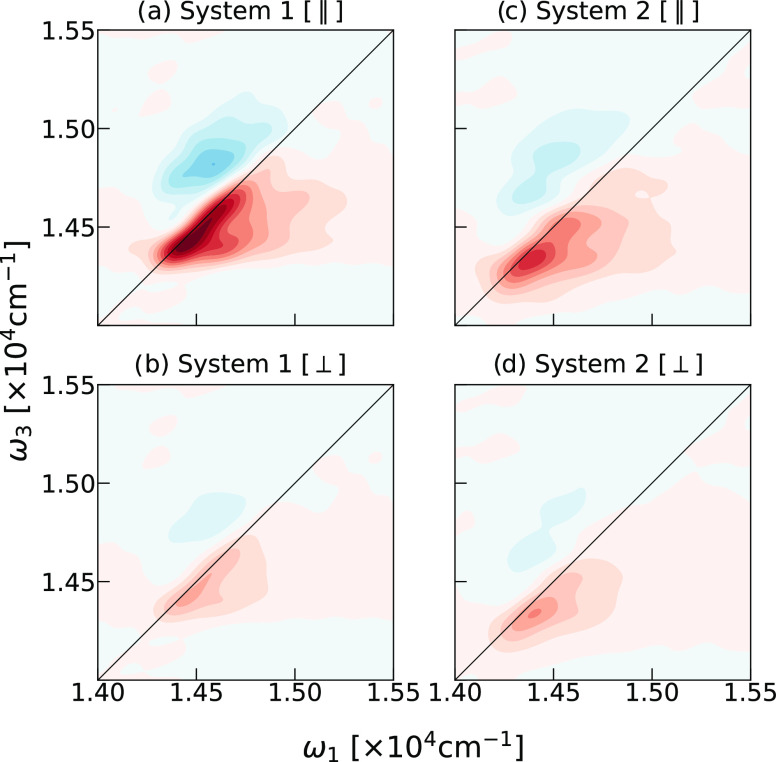
Comparison of 2D ES at time *t*_2_ = 0 for the two chlorosome model systems, as obtained with different ultrafast polarization configurations: (a, c) parallel polarization; (b, d) perpendicular polarization. Spectra of both systems are normalized with respect to the maximal absolute value of the signal in the parallel polarized spectra of each system.

Next, we examined the simulated 2D ES spectra of the two chlorosome model systems. The spectra were calculated for the two smaller subsystems with both parallel and perpendicularly polarized pulse sequences.^41,49^ We confirm that finite size effects are limited by comparing simulated linear absorption spectra of smaller cylinders to whole three-cylinder systems, as shown in Figure S4. We show the absorptive parts of the 2D ES spectra at zero waiting times (*t*_2_ = 0) in Figure 3. The observed spectral features agree very well with the experimental reports,^9,34,35^ supporting our simulation protocol. The diagonal peak, shown in red, displays contributions from single-excitation processes such as ground state bleach (GSB) and stimulated emission (SE). Since these processes capture the depletion of the population of the ground state and the enhanced emission from the excited state, their signals carry the same sign. The peak lying higher along the detection (ω_3_) axis arises from excited-state absorption (ESA). This process involved absorption into the manifold of double-excited states. Hence, the spectral peak carries an opposite sign compared to the GSB/SE peak and is represented by the blue color of the contour lines.

Significant elongation of the diagonal feature shows the correlation between the pump frequency and the detected signal, displaying substantial inhomogeneous broadening. Good agreement with experimental observations^9,34,35^ confirms the crucial role of molecular disorder in spectral broadening, as indicated by hole-burning studies^23^ and illustrated previously in ref (31), for System 1. In line with the linear absorption spectra, System 2 has a broader 2D ES than System 1 for both parallel and perpendicularly polarized spectra. Additionally, the perpendicularly polarized 2D ES of System 2 exhibits higher spectral intensity due to the absence of a preferential direction of the excitonic transition dipole moments, allowing for significant absorption in different spatial directions, as discussed previously.

**Figure 4:**
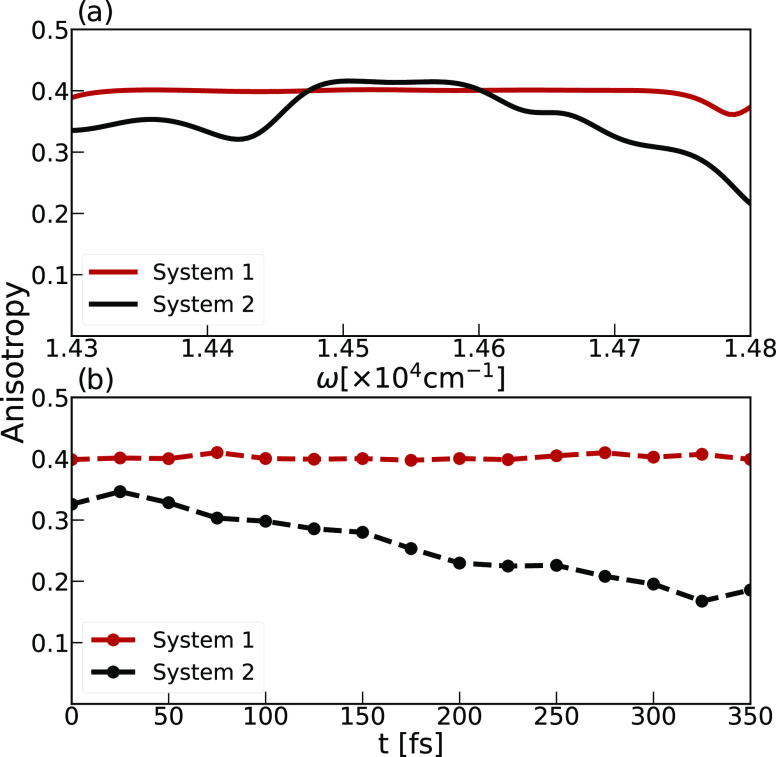
Anisotropy spectra (a) and anisotropy decay profiles (b) as estimated for two chlorosome model systems shown in red (System 1) and black (System 2). (a) Fundamental anisotropy spectrum is calculated for the points on the diagonal (ω_1_ = ω_3_) based on the polarized 2D ES spectra calculated for *t*_2_ = 0 waiting time. (b) Anisotropy decay profiles determined for the points in the 2D ES of maximal intensity: System 1 (ω_1_ = 14430 cm^–1^, ω_3_ = 14401 cm^–1^) and System 2 (ω_1_ = 14397 cm^–1^, ω_3_ = 14345 cm^–1^).

We will combine information from the polarized 2D ES with eq 2 to determine the anisotropy. At waiting time *t*_2_ = 0, we can neglect the effects of dynamical processes leading to reorientations of the transition dipole moments and estimate the values of the fundamental anisotropy *r*(0) and its dependence on the excitation energy, i.e., the anisotropy spectrum.^55^ The maximal value of *r*(0) of 0.4 corresponds to systems where transition dipole moments align with the main axis of the system.^55^ We note that spectral overlap of signals with opposite signs (GSB/SE vs ESA) introduces significant uncertainty in the estimated anisotropy for such points.^52^ Hence, we focus our analysis on the diagonal feature in the 2D ES and characterize populations of the single exciton states. The resulting anisotropy spectra of the two chlorosome model systems, as evaluated for the points at the diagonal (ω_1_ = ω_3_), are shown in Figure 4a. The anisotropy spectrum evaluated for System 1 shows minimal variations from *r*(0) ≈ 0.4, revealing an exciton band of states with predominant alignment of the transition dipole moments with the long axis. This finding aligns with the previous description of System 1 and with observations from transient absorption anisotropy measurements of chlorosomes isolated from *C. auranticus*(32) and samples from *C. tepidum* grown in higher light conditions.^57^

The anisotropy spectrum of System 2 is more complex. Significant variations are due to distinct spectral bands shown in Figure 2d, arising from exciton states with transition dipole moments noncollinear with the long axis of the aggregate. Such variations appear due to the spectral overlap with cross peaks^80^ coming from coherent excitations of states with different directions of transition dipole moments or by coherent transfer occurring during the coherence times *t*_1_ and *t*_3_.^58^ Since the *I*_⊥_ components are more sensitive to the length and radius of the cylinder, we provide information on spectral components in the small System 2 in Figure S5 in the Supporting Information. The lower energy part of the anisotropy spectrum, characterized by *r*(0) ≈ 0.3, confirms a significant contribution of both the *I*_∥_ and *I*_⊥_ components to the optical response. The anisotropy increases through the band, reaching its maximum of *r*(0) ≈ 0.4 in the domain of maximum absorption. Later, the anisotropy gradually decreases with the increased contributions of the *I*_⊥_ component to the total optical response. Notably, pump–probe experiments on chlorosomes from *C. tepidium*(29) reported the initial anisotropy values around *r*(0) ≈ 0.3.

From the information on the time-evolution of correlations in the polarization-resolved 2D ES, we determined the anisotropy decay profiles elucidating the nature of the underlying ultrafast exciton dynamics.^9,34,35,47,48^ We focus on the points with maximum absolute intensity in the 2D ES parallel spectra at *t*_2_ = 0, here (ω_1_ = 14430 cm^–1^, ω_3_ = 14401 cm^–1^) and (ω_1_ = 14397 cm^–1^, ω_3_ = 14345 cm^–1^) for Systems 1 and 2, respectively.

In System 1, exciton states contributing to the chosen point all have transition dipole moments with preferential orientation parallel to the cylinder axis, leading to *r*(0) = 0.4. This applies even when we have an isotropic collection of such cylinders as long as no exciton transfer between the cylinders takes place on this (*t*_2_ ≈ 0 fs) time scale.^80^ On the other hand, *r*(0) = 0.3 is evidence of the contribution of cross peaks between the states with different directions of transition dipole moments to the chosen point in the 2D ES spectrum of System 2.

Figure 4b shows a remarkable difference in anisotropy kinetics in two chlorosome model systems occurring during the first 350 fs. While System 1 does not exhibit anisotropy decay, System 2 manifests an ultrafast anisotropy decay on the time scale of τ ≈ 500 fs estimated from the exponential fit. Rotational diffusion is very restricted in chlorosomes,^19,31^ as in other molecular aggregates, so it can be ruled out as a source of the ultrafast anisotropy decay.

System 2 hosts exciton states exhibiting a broad distribution of orientation of transition dipole moments, as shown in Figure S2. Therefore, we confirm that the ultrafast anisotropy decay in System 2 arises from energy transfer between exciton states within the same cylinder that have significantly different directions of the transition dipole moments. Dynamic disorder breaks the symmetry of the cylinder and promotes transfer between different states (*k*_2_ = ±1 like) with transition dipole predominantly in the plane perpendicular to the cylinder axis. Furthermore, transfer between these states and (*k*_2_ = 0 like) states with the transition dipole parallel with the cylinder axis allows for an additional contribution to the observed ultrafast anisotropy decay.

Here, ultrafast mixing of excitonic states, enhanced by significant spectral overlap and large transition dipole moments, leads to the observed anisotropy decay as an outcome of supertransfer of excitons.^81,82^ In contrast, as exciton states in System 1 have the same polarization, energy transfer occurs between the states with the same directions of the transition dipole moment, so the value of anisotropy remains constant. In this case, we cannot say anything about the energy transfer processes occurring in this system.

With this, we demonstrate that ultrafast anisotropy decay experiments can capture the ultrafast transfer of excitons characterized by the lack of the preferential direction of the transition dipole moments. Furthermore, this experiment can provide a handle on determining the helicity in a given chlorosome and reconcile contrasting proposals for structures of chlorosomes based on different experiments.^20,27^ Localization of exciton states within the single aggregate due to the presence of disorder^9,31^ and the estimated distance between different cylinders in chlorosomes is 2.1 nm,^17^ ruling out exciton delocalization over multiple cylinders. Hence incoherent energy transfer between cylinders is expected to occur on a slower (≃1 ps) time scale.^5,33^ Since the time scale of the observed ultrafast anisotropy decay coincides with the exciton dynamics occurring within an individual aggregate,^9,30^ the anisotropy experiment is not affected by multiple aggregates with potentially different orientations that may be present in chlorosomes as long as there is no energy transfer between them on the examined subpicosecond time scale.^80^ The presence of cylinders with different orientations could influence conclusions in single-chlorosome experiments,^27^ which rely on the assumption that the cylinders are aligned with each other within the individual chlorosome and even more so in linear dichroism experiments, which rely on the alignment of all cylinders in the macroscopic sample. As an ensemble experiment, ultrafast anisotropy decay was performed on an isotropic sample. It does not require the alignment of chlorosomes, as in linear dichroism experiments. Since the helicity of chlorosomes may depend on growing conditions,^78^ bacterial species,^29^ and mutations,^27^ ultrafast anisotropy decay is a well-suited experiment to probe the helicity of specific samples. Finally, these experiments are much less complicated to interpret than circular dichroism experiments.

## Conclusions

In summary, we presented a comparative study of spectral properties of two atomistic structures of chlorosomes, consisting of cylindrical helical supramolecular aggregates that differ only in their helicity. Spectral properties of the model systems are in agreement with experimental observations for chlorosomes from green nonsulfur *C. auranticus*(25,28,32) and green sulfur bacteria *C. tepidum*.^9,25,27,28,33,34,36,79^ We demonstrated the intricate relationship between the helicity of the structures and the present excitonic states. These states are responsible for the system’s interaction with light^22^ and ensuring extremely efficient excitation energy transfer.^8,9^ The helicity dictates the arrangement of the BChl *c* molecules within the aggregates, and it tunes the absorption of light incoming from the parallel and perpendicular direction to the chlorosome, resulting in optical domains with different polarization properties, as observed in single-molecule spectroscopy experiments.^27,79^ We show how the helicity of cylindrical aggregates alters the overall spectral width and the ultrafast anisotropy decay. Hence, we demonstrate how a change in the molecular arrangement can optimize efficient light capture,^8^ suggesting alternation of the helicity of aggregates as a molecular mechanism allowing adaptation to the versatile light conditions. This mechanism is similar, or even coupled with the observed production of BChl homologues with longer side chains,^83,84^ which would significantly change the packing of the molecules within the aggregate. This argument is also consistent with observations of drastic changes in circular dichroism spectra of chlorosomes prepared with slightly different procedures.^25,85^

Furthermore, our simulated two-dimensional electronic spectra agree well with the experimental ones.^9,34^ Our study demonstrates the utility of the polarization-dependent 2D ES spectra for obtaining the structure information on cylindrical aggregates. These experiments are performed on an isotropic sample,^41^ overcoming difficulties of linear dichroism requiring oriented samples or extensive time-demand necessary for signal acquisition in the single-molecule experiments.^16,27,57^ Previously, we established a relationship between different roles of BChl *c* molecules in hydrogen bonding with exciton delocalization and the presence of distinct optical domains^31^ that clarifies the idea of coherent domains^9^ between which observed subpicosecond ultrafast energy transfer occurs. Presented calculations of polarization-resolved 2D ES and anisotropy decay profiles uncovered the contrasting mechanisms that underpin ultrafast energy transfer pathways present in chlorosome systems with different helicities. This study opens possibilities for future research on the role of molecular noise^59,86^ and coherent beatings^9,33^ on the observed ultrafast exciton transfer based on the microscopic model. Additionally, our findings can inspire novel ideas for light capture and energy transfer in artificial light-harvesting systems and organic photovoltaics.^8,11,14,15^

## Supporting Information Available

The Supporting Information is available free of charge at: DOI. The detailed description of the parameters used for the constructed structures and comparison of the spectral properties for tube systems of different sizes (PDF)
