# Supplementary material for: Ultrafast Anisotropy Decay Reveals Structure and Energy Transfer in Supramolecular Aggregates
Source: J Phys Chem B. 2023 Aug 18;127(34):7487–96. doi: 10.1021/acs.jpcb.3c04719 (PMC10476209; doi:10.1021/acs.jpcb.3c04719)
Supplement: Supplementary file 1 — jp3c04719_si_001.pdf [file jp3c04719_si_001.pdf]

Supplementary Information for: Ultrafast Anisotropy Decay Reveals Structure and Energy Transfer in Supramolecular Aggregates

Vesna Erić,† Jorge Luis Castro,† Xinmeng Li,‡ Lolita Dsouza,¶ Sean K. Frehan,§ Annemarie Huijser,§ Alfred R.Holzwarth,‖ Francesco Buda,¶ G. J. Agur Sevink,¶ Huub J. M. de Groot,¶ and Thomas L. C. Jansen*,†

†*University of Groningen, Zernike Institute for Advanced Materials, 9747 AG Groningen, The Netherlands.*

‡*Department of Chemistry and Hylleraas Centre for Quantum Molecular Sciences, University of Oslo, Sem Sælands vei 26, 0315 Oslo, Norway*

¶*Leiden Institute of Chemistry, Leiden University, Einsteinweg 55, 2300 RA Leiden, the Netherlands*

§*MESA+ Institute for Nanotechnology, University of Twente, Drienerlolaan 5, 7522 NB Enschede, the Netherlands*

‖*Department of Biophysical Chemistry, Max Planck Institute for Chemical Energy Conversion, Stiftstraße 34-36, 45470 Mülheim, Germany*

E-mail: t.l.c.jansen@rug.nl

Initial structure

Initial structures of chlorosome model systems are created by rolling up a 2D sheet structure into cylinders. Lattice parameters such as vectors $\vec{a}$ and $\vec{b}$ and the angle between them γ

giving information on the unit cell are obtained from solid state NMR studies[1] and are (a, b, γ)=(1.48 nm, 0.98 nm, 124.3^o). A schematic representation of the 2D lattice is given in Fig S1.

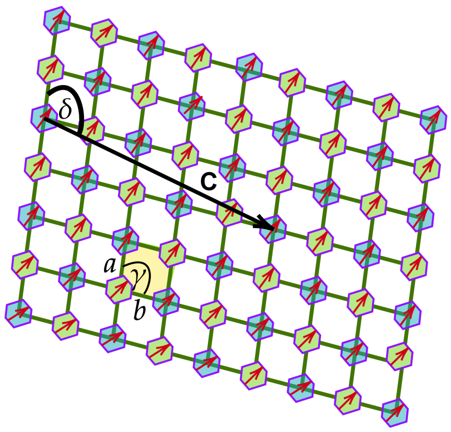

Figure S1: Schematic representation of a 2D sheet which is rolled along the chiral vector C into the tube, following the procedure explained in Refs. 2–5.

We used the software CTubeGen[6] to generate the two model systems represented as three concentric cylinders, that are defined by their radius R and the chiral angle δ. Three cylinders will be referred to as inner, middle and outer cylinder. Parameters describing two model systems are summarized in Table S1.

Table S1: Characterization of the linear absorption spectra of small model system for which simulations of 2D electronic spectra are performed.

Initial structure parameters		
	System 1	System 2
Number of molecules	27675	27829
$\delta(^o)$	49.5	112.3
R_{inner}(nm)	5.2	4.7
R_{middle}(nm)	7.3	7.4
R_{outer} (nm)	9.5	10.2

Transition dipole moments of exciton states

In the main text, we discussed the impact of the molecular packing within chlorosomes, with a focus on the angle β, the angle between the molecular transition dipole moment μ and the cylinder axis of the chlorosome, on the optical response of the system. This angle will dictate the orientation of the transition dipole moments of exciton states, $\mu_{exciton}$. A large number of the optically active excitons in cylindrical aggregates arise due to the presence of structural disorder included in our model. In Fig. S2 we give the angles that individual exciton transition dipole moments, $\mu_{exciton}$, form with the respective coordinate axes z (θ), $x(\phi_x)$, and $y(\phi_y)$.

Since we are mostly interested in optically active, superradiant, excitons: we show the dependence of the oscillator strength $\mu^2_{exciton}$ on these angles. While in System 1 the transition dipole moments clearly preferentially orient in the direction of the cylinder axis, a clear preferential direction is absent in System 2. In line with the discussion in the main text, we thus see that the bright exciton states in System 1, are predominantly almost parallel to the long z axis. In contrast, in System 2, the distribution of orientations of the exciton transition dipole moments, $\mu_{exciton}$, is much broader. This is the fundamental background leading to the ultrafast anisotropy decay that we report for this system.

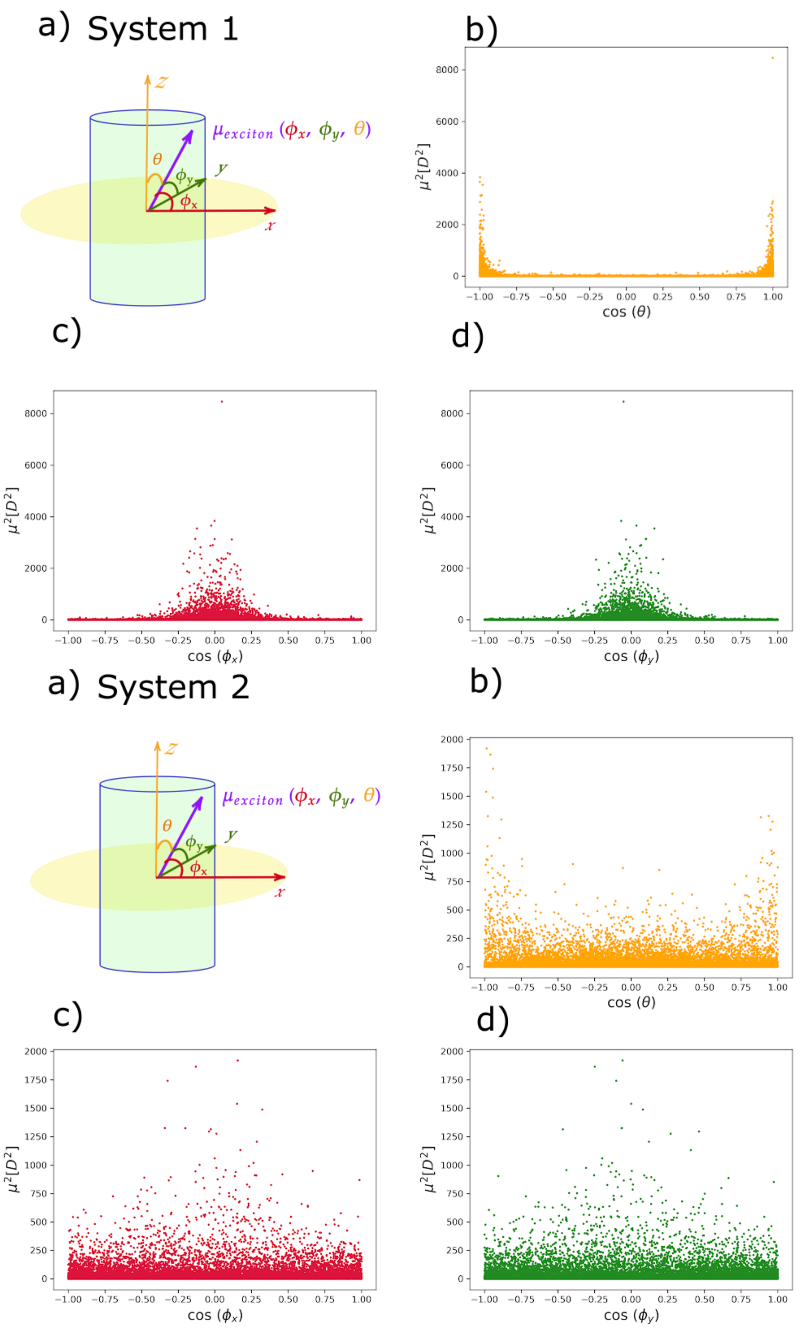

Figure S2: Characterization of the orientation of the transition dipole moments $\mu_{exciton}$ in System 1 (left panel) and System 2 (right panel). a) Representation of the coordinate system where z is chosen as a long axis of the chlorosome cylinder, while x and y define the xy plane perpendicular to z. Transition dipole moment vectors of exciton states enclose angle θ with the long axis and angles ϕ_x and ϕ_y with the x and y, respectively. b), c), d) show the dependence of the oscillator strength $\mu^2_{exciton}$ on the angle that transition dipole moments enclose with respective coordinates z, x, y: $\cos\theta$ (in orange), $\cos\phi_x$ (in red) and $\cos\phi_y$ (in green), respectively.

Comparison of simulated to experimental linear absorption spectra

In Fig. S3 we compare our simulated linear absorption spectra of System 1 and System 2 with the experimentally measured spectra for *Chloroflexus aurantiacus*[7] and *Chlorobaculum tepidium*.[8] For the most direct comparison with the experiment, we included effects of homogeneous and inhomogeneous broadening, caused by the presence of mesoscale disorder[9] on the linear absorption and 2D ES spectra by weighting simulated response functions with exponential (τ_{homo} = 300 fs) and Gaussian (τ_{inhomo} = 166 fs) apodization functions,[10] as reported previously in Ref. 11. We note that local structural disorder and bath fluctuations, as included in our model, are responsible for the resulting spectral width, while the convolution procedure leads to smoother spectra, but no significant additional broadening. All spectra are normalized to the maximal peak height. The spectra of System 1 and System 2 are red-shifted by 1150 cm^{-1} and 1350 cm^{-1}, respectively. The required red-shift may be explained by a combination of the choice of the frequency of the isolated chromophores[3,11] and the size of the couplings predicted by the transition dipole coupling model.[3,11] The low intensity absorption band centered around 12500 cm^{-1} in the spectrum of *Chloroflexus aurantiacus* comes from BChl *a* of the baseplate. Likewise there is a contribution of the baseplate absorption in the same frequency region in the spectrum of *Chlorobaculum tepidium* effectively broadening the spectrum. Our model systems do not include baseplate and, thus, do not include these features. The comparisons confirm that System 1 and System 2 are good models of chlorosomes from *Chloroflexus aurantiacus* and *Chlorobaculum tepidium*, respectively.

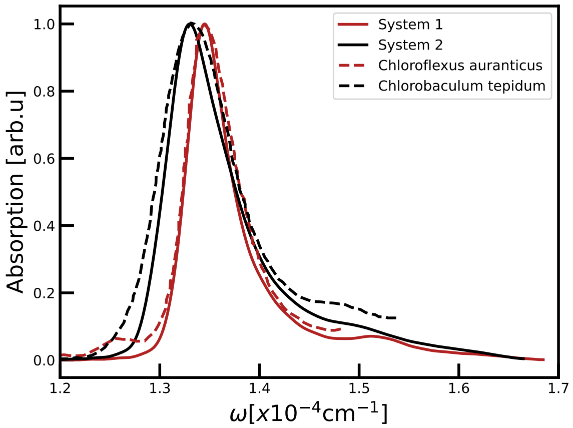

Figure S3: Comparison of the simulated linear absorption spectra of System 1 (red) and System 2 (black) with experimental spectra of *Chloroflexus aurantiacus*[7] (red dashed) and *Chlorobaculum tepidium*[8] (black dashed). The experimental spectra for chlorosomes for the two bacterial species are digitized from Refs. 7 and 8.

Characterization of the small model that is used for calculation of 2D ES spectra

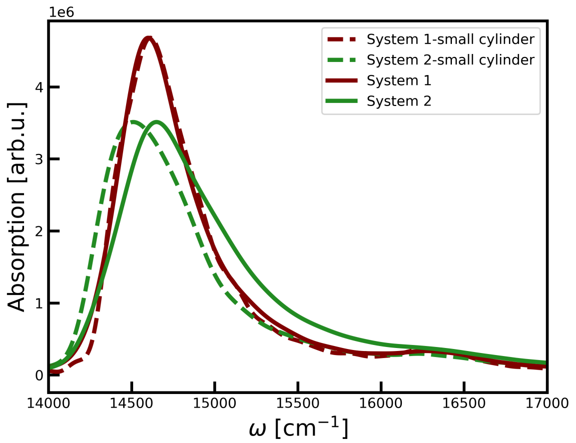

Figure S4: Comparison of linear absorption spectra calculated for small cylindrical systems, used for calculations of 2D ES of the two chlorosome models System 1 (red) and System 2 (green).

Table S2: Characterization of the linear absorption spectra of a small model system for which simulation of 2D ES are performed. The peak positions and FWHM were extracted directly from the spectra presented in Fig. S4, which were calculated using a quantum-classical simulation procedure[12] based on the exciton model including the structural disorder predicted from the molecular dynamics simulations as well as the discussed mesoscopic disorder.

Linear absorption spectra			
	Number of molecules	$\langle\omega\rangle$ (cm^{-1})	FWHM (cm^{-1})
System 1	2639	14614	550
System 2	2675	14512	664

We observed that the absorption spectrum of the small model of System 2 is additionally shifted towards the red compared to the spectra of the full system. The decomposition of these spectra on components which define intensity in the direction parallel and perpendicular to the long axis of the cylinder is shown in Figure S5. Based on this we correlate the additional shift to the shift of high energy (perpendicularly polarized) exciton states,

which strongly depend on the radius of the tube.[13] Information on peak positions and full-width-half-maximum of two spectral components estimated for the small model representing System 2 are given in the Table S3. Presented information is useful for better understanding of peak positions in calculated 2D spectra shown in Figure 3. and anisotropy spectrum in Figure 4. that are given in the main text.

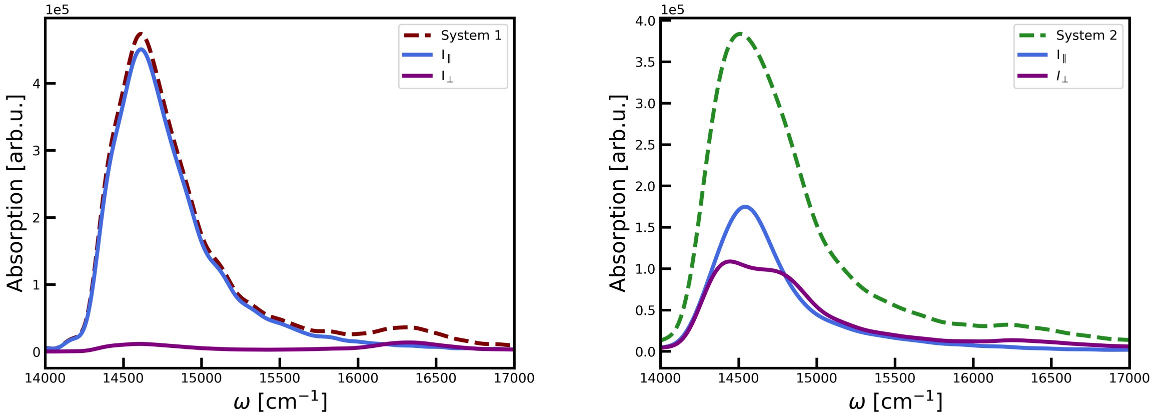

Figure S5: Decomposition of linear absorption spectra calculated for the small chlorosome model systems representing System 1 (left panel) and System 2 (right panel) on parallel $I_{\parallel}$ and perpendicular components $I_{\perp}$.

Table S3: Parameters describing positions and widths of parallel and perpendicular spectral component of the small model representing System 2.

Linear absorption spectra decomposition		
	$\langle\omega\rangle(\mathrm{cm}^{-1})$	FWHM (cm^{-1})
$I_{\parallel}$	14544	505
$I_{\perp}$	14466	754

References

(1) Ganapathy, S.; Oostergetel, G. T.; Wawrzyniak, P. K.; Reus, M.; Gomez Maqueo Chew, A.; Buda, F.; Boekema, E. J.; Bryant, D. A.; Holzwarth, A. R.;

De Groot, H. J. Alternating syn-anti bacteriochlorophylls form concentric helical nanotubes in chlorosomes. *Proc. Natl. Acad. Sci.* **2009**, *106*, 8525–8530.

(2) Li, X.; Buda, F.; de Groot, H. J.; Sevink, G. A. Contrasting modes of self-assembly and hydrogen-bonding heterogeneity in chlorosomes of Chlorobaculum tepidum. *J. Phys. Chem. C* **2018**, *122*, 14877–14888.

(3) Li, X.; Buda, F.; de Groot, H. J.; Sevink, G. A. Molecular insight in the optical response of tubular chlorosomal assemblies. *J. Phys. Chem. C* **2019**, *123*, 16462–16478.

(4) Günther, L. M.; Jendrny, M.; Bloemsma, E. A.; Tank, M.; Oostergetel, G. T.; Bryant, D. A.; Knoester, J.; Köhler, J. Structure of light-harvesting aggregates in individual chlorosomes. *J. Phys. Chem. B.* **2016**, *120*, 5367–5376.

(5) Günther, L. M.; Löhner, A.; Reiher, C.; Kunsel, T.; Jansen, T. L. C.; Tank, M.; Bryant, D. A.; Knoester, J.; Köhler, J. Structural variations in chlorosomes from wild-type and a bchQR mutant of Chlorobaculum tepidum revealed by single-molecule spectroscopy. *J. Phys. Chem. B* **2018**, *122*, 6712–6723.

(6) Li, X.; Buda, F.; de Groot, H. J.; Sevink, G. A. The role of chirality and plastic crystallinity in the optical and mechanical properties of chlorosomes. *Iscience* **2022**, *25*, 103618.

(7) Martiskainen, J.; Linnanto, J.; Kananavičius, R.; Lehtovuori, V.; Korppi-Tommola, J. Excitation energy transfer in isolated chlorosomes from Chloroflexus aurantiacus. *Chem. Phys. Lett.* **2009**, *477*, 216–220.

(8) Martiskainen, J.; Linnanto, J.; Aumanen, V.; Myllyperkiö, P.; Korppi-Tommola, J. Excitation energy transfer in isolated chlorosomes from Chlorobaculum tepidum and Prosthecochloris aestuarii. *Photochem. Photobiol.* **2012**, *88*, 675–683.

(9) Furumaki, S.; Vacha, F.; Habuchi, S.; Tsukatani, Y.; Bryant, D. A.; Vacha, M. Absorption linear dichroism measured directly on a single light-harvesting system: the role of disorder in chlorosomes of green photosynthetic bacteria. *J. Am. Chem. Soc.* **2011**, *133*, 6703–6710.

(10) Hamm, P.; Zanni, M. *Concepts and methods of 2D infrared spectroscopy*; Cambridge University Press, 2011.

(11) Erić, V.; Li, X.; Dsouza, L.; Frehan, S. K.; Huijser, A.; Holzwarth, A. R.; Buda, F.; Sevink, G. A.; de Groot, H. J.; Jansen, T. L. C. Manifestation of Hydrogen Bonding and Exciton Delocalization on the Absorption and Two-Dimensional Electronic Spectra of Chlorosomes. *J. Phys. Chem. B* **2023**, *127*, 1097–1109.

(12) Jansen, T. L. C. Computational spectroscopy of complex systems. *J. Chem. Phys* **2021**, *155*, 170901.

(13) Bondarenko, A. S.; Jansen, T. L. C.; Knoester, J. Exciton localization in tubular molecular aggregates: Size effects and optical response. *J. Chem. Phys* **2020**, *152*, 194302.
